# Colostrum and Lactoferrin Protect against Side Effects of Therapy with Antibiotics, Anti-inflammatory Drugs and Steroids, and Psychophysical Stress: A Comprehensive Review

**DOI:** 10.3390/biomedicines11041015

**Published:** 2023-03-27

**Authors:** Jolanta Artym, Michał Zimecki

**Affiliations:** Department of Experimental Therapy, Hirszfeld Institute of Immunology and Experimental Therapy, Polish Academy of Sciences, R. Weigla 12 Str., 53-114 Wroclaw, Poland

**Keywords:** lactoferrin, bovine colostrum, adverse side effects, NSAIDs, antibiotic therapy, synthetic corticosteroids, endogenous corticosteroids, psychophysical stress

## Abstract

In this article, we review the benefits of applying bovine colostrum (BC) and lactoferrin (LF) in animal models and clinical trials that include corticosteroid application and psychic stress, treatment with non-steroid anti-inflammatory drugs (NSAIDs) and antibiotics. A majority of the reported investigations were performed with native bovine or recombinant human LF, applied alone or in combination with probiotics, as nutraceutics and diet supplements. Apart from reducing adverse side effects of the applied therapeutics, BC and LF augmented their efficacy and improved the wellness of patients. In conclusion, LF and complete native colostrum, preferably administered with probiotic bacteria, are highly recommended for inclusion in therapeutic protocols in NSAIDs and corticosteroid anti-inflammatory, as well as antibiotic, therapies. These colostrum-based products can also be of value for individuals subjected to prolonged psychophysical stress (mediated by endogenous corticosteroids), especially at high ambient temperatures (soldiers and emergency services), as well as physically active people and training athletes. They are also recommended for patients during recovery from trauma and surgery, which are always associated with severe psychophysical stress.

## 1. Introduction

The sequential introduction of chemotherapeutics, such as antimetabolites, sulfonamides and antibiotics, as well as nonsteroid anti-inflammatory drugs (NSAIDs) and synthetic corticosteroids, to therapy at the beginning of the 20th century led to a breakthrough in combating neoplastic, infectious and inflammatory diseases [1,2,3,4]. The application of antimetabolite chemotherapeutics, such as aminopterin, cyclophosphamide and methotrexate, proved effective in treating cancer patients [2]. Sulfonamides and antibiotics are natural and synthetic substances that inhibit bacterial growth. First used on a large scale during the Second World War, they saved millions of lives. A number of deadly infectious diseases, such as pneumonia, tuberculosis, diphtheria, puerperal fever, syphilis, cholera and purulent wound infections, have been drastically eliminated [3]. Lastly, we witness serious problems associated with overuse of antibiotic therapy leading to antibiotic resistance and changes to physiologic processes and the microbiome [5]. It is also worth noting the discovery of bacteriophages, which were applied to treat bacterial infections before the antibiotic era and can now be a valuable alternative to antibiotics in the treatment of antibiotic-resistant strains of bacteria [6]. The first chemical drugs (antipyrin, phenacetin and salicylic acid derivatives) to relieve pain, lower body temperature and ameliorate inflammation appeared even earlier, at the end of the 19th century. In the years that followed, new drugs, exhibiting similar effects, were developed to treat many inflammatory diseases, including autoimmune ones [1]. Nowadays, the most commonly used anti-inflammatory, antipyretic and analgesic drugs are NSAIDs. In the form of oral and topical preparations, they are available without a prescription and are very often abused. Synthetic corticosteroids (glucocorticosteroids and steroids) are effective for similar therapeutic indications. Sometimes, they are also used as immunosuppressants or anti-allergic drugs. First used in the mid-1950s, they brought relief to a patient suffering from arthritis, hospitalized at the Mayo Clinic in Rochester, USA [4]. Endogenous steroids, released upon acute and chronic psychophysical stress serve to maintain the body’s homeostasis, but may also lead to undesirable physiological effects, in the immune system, among other things [7,8,9,10].

**Figure 1:**
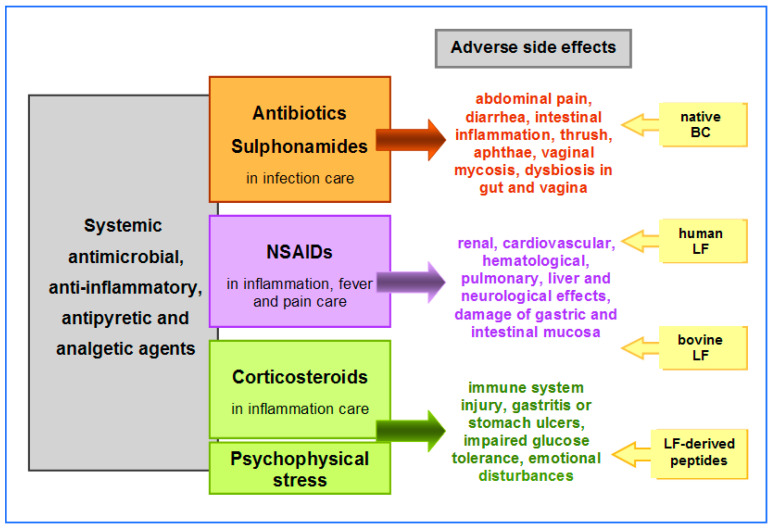
Systemic antimicrobial agents—antibiotics and sulfonamides and anti-inflammatory, antipyretic and analgetic agents—NSAIDs and synthetic corticosteroids in therapy of infections, inflammation, fever and pain. BC and its bioactive constituents (LF and other proteins) may be used as supportive care in preventing and treating adverse side effects of these therapies, as well as for the alleviation of endogenous, steroid-induced physiological effects of psychophysical stress.

All these categories of drugs, although of high therapeutic efficacy, are toxic to the patient’s tissues, because of their mechanisms of action. Usually, the immune and nervous systems and the gastrointestinal tract, as well as the physiological microbiota of the gut and the reproductive tract, are damaged. Adverse side effects of antibiotics/sulfonamides, NSAIDs and synthetic steroids, as well as endogenous steroids, in stress are summarized in Figure 1. Adverse effects may affect the whole organism or individual tissues and organs of the treated patient. They diminish the compliance and effectiveness of the therapy or even prevent a complete cure. In addition, they significantly impair the patient’s well-being and quality of life. Therefore, effective protective treatment is often as important as the primary therapy.

Thus, new agents are in high demand to improve the efficacy of the basic therapeutic protocols and lower their adverse side effects. Natural products—bovine colostrum (BC) and preparations containing its active ingredients, mainly lactoferrin (LF)—appeared to be suitable for this purpose. They exert multidirectional effects on the organism, are bioavailable, non-toxic, safe and easy to self-apply (as diet supplements and nutraceutics), and are relatively inexpensive. The benefits of applying BC, LF and its peptides as adjunct supportive care for cancer chemo- and radiation therapies were reviewed by us very recently [11].

This article reviews the use of these products as adjuncts to therapy with antibiotics, NSAIDs and synthetic steroids and in subjects exposed to psychophysical stress, in animal and human models. It represents the completion of our previous article on the application of BC and LF in cancer chemo- and radiotherapy [11].

## 2. Bovine Colostrum

After birth, the growth, maturation and protection of newborn mammals are supported by breast milk. Apart from nutrients (proteins, fats, minerals and vitamins), it contains numerous enzymes, hormones, growth factors, cytokines, immune cells and immunoglobulins, and even symbiotic bacteria that slowly colonize the newborn’s body. The most valuable milk for newborns is that produced during the first few days (3–4) after birth, i.e., colostrum, also referred to as initial or first milk. So far, about 250 of the active constituents of this milk have been identified, although undoubtedly many still await discovery and characterization of their activities. Colostrum is a thick, yellowish and oily substance produced by the secretory epithelial cells of the mammary gland. Although it is produced in small quantities, due to its wealth of nutritional, regulatory and protective components, it is sufficient to meet the needs of the newborn baby. Colostrum ensures the proper development of all the baby’s tissues and organs, especially the nervous, digestive and immune systems. The first milk also protects the susceptible organism of the newborn from various types of infections, especially those encountered by the mother [12,13]. A colostrum-fed newborn is protected particularly effectively against infection and other gastrointestinal disorders because the active ingredients in colostrum act directly on its tissues. The nutrients and regulatory substances ensure normal growth, differentiation, maturation and function of the digestive system, protect against damage and allow it to heal, control the development of a normal intestinal microbiota and shape the local (gut-related) and systemic immune response.

Being aware of so many benefits of colostrum for the newborn baby, people have learned to use human and animal colostrum in preventing and treating various infirmities and diseases in older children and adults as well. Owing to its availability, BC has been the most widely used for thousands of years. Applied in folk medicine, it has healed wounds, protected against infections and helped convalescents. Nowadays, BC, as a natural source of nutritional, regulatory and defensive components, is increasingly appreciated also in academic medicine. It is widely recommended for children and adults to prevent and treat various disorders and diseases. Numerous preclinical and clinical studies have demonstrated its efficacy in the prevention and treatment of respiratory and gastrointestinal infections, urogenital conditions, normalization of carbohydrate and lipid metabolism, wound healing, regeneration, sport and anti-ageing medicine [14,15,16,17,18,19,20,21,22].

Due to its availability and relatively low price, it is mainly BC that is currently used worldwide, although goat and horse colostrum are also available. It is used as a food collected from cows in traditional products of national cuisines (cheese, curd, fermented colostrum and colostrum desserts). Ultrafiltrated BC product as powder is widely used as a dietary supplement (powders, capsules, lozenges and chewable tablets), functional food additive (dairy drinks, yoghurt, kefir, ice cream and fruit jellies) and as an ingredient in cosmetics (skin cream, skin moisturizers and skin emulsion) [15,16,23,24]. BC is also used as a feed additive in cattle, pig, poultry and fish farming. This practice has resulted in increases in survival rates and improvement in the overall health of animals [16,25,26].

**Figure 2:**
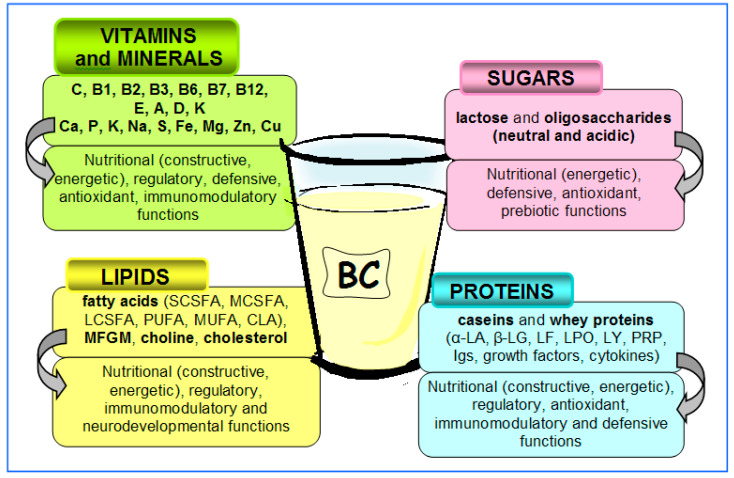
Selected macronutrients and micronutrients present in bovine colostrum. They ensure the nutritional value of colostrum (build up the body and provide energy), regulate metabolic processes, protect against pathogens and oxidative stress, and ensure correct development of the nervous system, digestive system and physiological symbiotic microbiota. α-LA—α-lactoalbumin, β-lactoglobulin, LF—lactoferrin, LPO—lactoperoxidase, LY—lysozyme, PRP—proline rich polypeptide (colostrinin), MFGM—milk fat globule membrane (protein and fat (mainly gangliosides and phospholipids) composition), SCSFA—short-chain saturated fatty acid, MCSFA—medium-chain saturated fatty acid, LCSFA—long-chain saturated fatty acid, PUFA—polyunsaturated fatty acid, MUFA—monounsaturated fatty acid, CLA—conjugated linoleic acid, C—vitamin C (ascorbic acid), B1—thiamine, B2 (riboflavin), B3—niacin, B6—pyridoxal, B7—biotin, B12—cobalamin, E—tocoferol, A—retinol, D—cholecalciferol, K—phylloquinone, Ca—calcium, P—phosphorous, Na—sodium, K—potassium, S—sulfur, Zn—zinc, Cu—copper, Fe—iron, Mg—magnesium.

An overview of BC components and its activity is presented in Figure 2 and in detail in our recent article [11]. The most important components of BC are briefly discussed here [14,15,17,20], including:

(1) Nutrients: building proteins (caseins, α-lactoalbumin (α-LA), β-lactoglobulin/β-LG/), amino acids, milk sugar (lactose), fats (triglycerides), fatty acids, minerals and vitamins; their purpose is to provide energy and building materials for the fast-growing organism and to regulate the various metabolic processes;
(2) Antimicrobial and immunoregulatory factors: immunoglobulins (Igs), lactoferrin (LF), lactoperoxidase (LPO), lysozyme (LY), antioxidant substances, nucleotides/nucleosides, gangliosides, oligosaccharides/glycoconjugates, phospholipids, colostrinin (proline rich polypeptide (PRP)), cytokines and maternal leukocytes; their purpose is to directly destroy pathogenic microorganisms, promote the growth of symbiotic (beneficial) microorganisms and regulate the maturation and function of the immune system;
(3) Growth factors: insulin-like growth factor (IGF), epidermal growth factor (EGF), transforming growth factor (TGF), platelet-derived growth factor (PDGF), vascular endothelial growth factor (VEGF) and hormones (prolactin, calcitonin, thyroxine, insulin and growth hormone); their function is to regulate the growth, maturation and function of various tissues, such as bone, muscle, connective tissue, nerve tissue, skin and especially the gastrointestinal mucosa; they act both locally (in the intestine) and systemically, after absorption into the circulation.

Administered orally, the components of BC directly affect the tissues of the mouth and throat (tonsils and lymphatic tissue as a component of Waldeyer’s ring) and the gastrointestinal tract, including immune cells. Consequently, BC is most effective in a variety of upper respiratory and gastrointestinal conditions.

BC, hyperimmune BC (HBC—produced by immunizing cows with a specific pathogen before lactation begins) and specific Igs isolated from HBC are effective in the prevention and treatment of respiratory and gastrointestinal infections in children of all ages, including infants [19,27]. In a Turkish RCT involving 31 children aged 5–17 years with IgA deficiency, sucking lozenges with BC and LY alleviated viral upper respiratory tract infections (URTIs) [28]. In an Italian retrospective observational study involving 167 children (3–7 years), a formulation with BC and a probiotic reduced the number of URTIs requiring antibiotic therapy [29]. BC also relieved symptoms of inhalant allergy/asthma in 38 children in the Philippines [30].

BC, HBC and HBC-derived Igs administered to children with rotavirus infection improved their clinical condition. The treatment reduced the intensity of diarrhea (number of stools) and shortened its duration and the period of virus excretion from the body [31,32,33]. Meanwhile, in children infected with *Escherichia coli* or *Shigella*, a reduction in the frequency of bowel movements and vomiting, faster elimination of pathogens from the body and a shorter period of necessary hospitalization were observed after the application of these preparations [34,35]. A 2019 meta-analysis that included five RCTs in children with diarrhea and confirmed rotavirus or *E. coli* infection (total n = 324) demonstrated the efficacy of BC, HBC and Igs isolated from HBC. A milder course and faster resolution of diarrhea were reported, as well as a more efficient elimination of pathogens from the system [36]. Additionally, a recent blinded RCT conducted in Egypt involving 160 children aged 6 months to 2 years demonstrated the efficacy of BC in the treatment of acute diarrhea caused by rotavirus or pathogenic strains of *E. coli* or concurrent infection with these pathogens [37]. In another trial, diet supplementation with BC and egg powder over 3 months improved growth rates in 277 infants in Malawi and alleviated intestinal dysfunction (improved intestinal epithelial tightness), but did not change the composition of the intestinal microbiota or the incidence of diarrhea [38].

Preclinical and clinical studies also suggest that BC may prevent cases of necrotizing enterocolitis (NEC), infection and sepsis in preterm infants [19,39]. After 5 weeks of BC or BC and probiotic supplementation, children with autism (n = 8) reported fewer gastrointestinal symptoms (including diarrhea and perceived abdominal pain). Positive changes in children’s behavior (fewer episodes of irritability, stereotypies, lethargy or hyperactivity) were also observed [40]. In an RCT involving 62 Danish children with acute lymphoblastic leukemia (ALL) and treated with chemotherapy, BC alleviated mucositis [41].

BC and related preparations were also effective when used by adults. When applied topically, they relieved urogenital complaints in women [27,42]; prevented and alleviated URTIs and gastrointestinal infections, as well as inflammatory bowel disease [18,43,44,45,46,47]; protected against gastrointestinal damage after NSAID therapy [48]; and healed wounds, ulcers and skin lesions in allergic and autoimmune disorders [49]. BC could also be helpful in the management of nonorganic failure to thrive in children from poor developing countries and in controlling glucose and lipid metabolism in diabetic patients [22]. In critically ill patients, BC had a beneficial effect on intestinal permeability and gastrointestinal complications [27,50]. Lastly, in physically active people and competitive athletes, BC protected against the effects of psychophysical stress, such as weakened immunity, increased URTIs and gastrointestinal symptoms due to increased intestinal permeability [27,51,52,53,54].

From the numerous preclinical (in vitro and animal) and clinical trials conducted to date, a mechanism of beneficial effect of BC has emerged [14,15,17,45,49,55]. It represents the sum of the activities of its individual components, such as oligosaccharides, fatty acids, LF, LY, LPO, PRP, cytokines, chemokines, numerous growth factors, hormones, vitamins and minerals. Many of these substances act synergistically, that is, they enhance each other’s effects (for example, LF, LPO and LY inhibit microbial growth more strongly than when used alone; similarly, the effectiveness of LF and IgA, as well as LF and α-LA, is higher when used together) [15,18,19].

These substances have microbiostatic and microbicidal effects on all groups of microorganisms: bacteria, fungi, parasites and viruses. They can directly damage the cellular structures of bacteria, fungi and parasites and inhibit the entry of viruses into host cells and their replication. BC components regulate the immune response by its enhancement or suppression (depending on the organism’s current needs), inhibit infection and attenuate inflammatory reactions. They also exert a direct cytotoxic effect on tumor cells and enhance the anti-tumor immune response. Other properties of BC include regulation of metabolic processes, such as blood cell formation, iron absorption and storage, glucose and lipid metabolism, bone and cartilage formation and resorption, and wound healing of skin and mucous membranes. BC preparations have, in addition, beneficial effects on the tissues of the nervous system, respiratory system, gastrointestinal tract and genitourinary system. Their benefits in the digestive tract include, but are not limited to, renewing and protecting the intestinal epithelium, sealing the intestinal barrier, inhibiting bacterial overgrowth and neutralizing bacterial toxins, inhibiting viral infections, reducing inflammation and normalizing the intestinal microbiota [44,45]. The molecular mechanism of activity of BC components includes regulation of redox processes, cell receptors and signaling pathway activities and cytokine gene and other pro- and anti-inflammatory factors, as well as regulation of symbiotic gut microbiota [14,15,17,20,56,57,58,59,60,61,62,63,64,65,66,67,68,69,70].

Importantly, BC and BC-derived products (HBC and HBC-derived specific Igs) are natural and safe for patients in human and veterinary medicine. Toxicological investigations in experimental animals proved the safety of high doses of BC products used by gavage (doses up to 4200 mg/kg bw/day) and supplemented in normal diets (up to 10% content) [71,72]. In numerous preclinical and clinical studies, BC and related products were safe when administered orally (systemic effects) and topically (on mucous membranes and skin). They can be used by people of all ages and health conditions [19,27,44,45,46,47,49,52,53,54].

The aforementioned actions of the bioactive components of BC themselves protect against the development of various diseases and disorders, such as infections, cancers, inflammatory diseases (including autoimmune and allergic diseases), caries and periodontal disease, iron deficiency anemia, obesity/diabetes/hypertension (metabolic syndrome) and complications in healing wounds, bone fractures and injuries. In addition, BC administered to patients can accelerate the treatment of these conditions or support the action of standard therapies, including those with cytostatic agents, antibiotics, NSAIDs and synthetic steroids. The synergistic action with the applied drugs can lower the necessary dose or shorten the time of application, thus reducing their toxicity for a patient. In conclusion, BC can mitigate adverse side effects of the undertaken therapy, improve compliance and ultimately improve the efficacy of the treatment.

## 3. Lactoferrin

To date, the best-studied active ingredient in BC and milk is LF. It is an evolutionarily old protein present in excretory fluids of mammals and secondary granules of neutrophils and is released from them at sites of inflammation. This 80 KDa, single polypeptide chain protein belongs to the transferring family and has a property to bind two Fe^3+^ ions. Lactoferrins from various species have similar amino acid sequences and similar tertiary structures. This high interspecies homology indicates a similar function for this protein in different mammalian species [60,65,66,73,74].

Over the past 60 years of intensive research, as many as 20 different physiological activities of LF have been described [73,74,75,76,77,78]. In this article, we will mainly focus on LF, with particular attention being paid to its actions in the amelioration of side effects caused by antibiotic and NSAID therapies, as well as psychophysical stress, so LF activity is described below in some detail.

**Figure 3:**
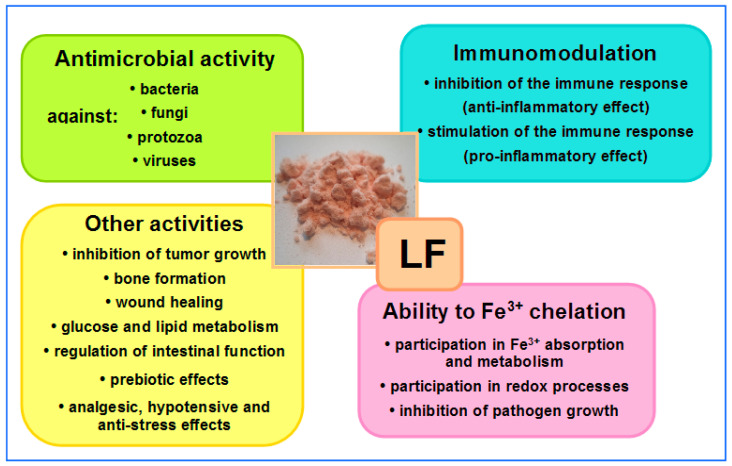
Overview of LF activities. In the central part, native LF isolated from bovine milk—salmon-colored powder (the intensity of the color depends on the degree of iron saturation—the more iron bound to the LF molecules, the darker the color of the powder); native LF is saturated at around 10% (photo by J. Artym).

The ability to bind iron, fight microbes and regulate hematopoiesis are among the most important properties of LF and LF-derived peptides. Other properties of LF include regulating immune system function (immunosuppression or immunoactivation) and oxidoreduction (redox) processes (i.e., those during which oxygen free radicals are released), inhibiting cancer cell growth, promoting osteogenesis and wound healing, regulating glucose and lipid metabolism, supporting bowel functions and enriching intestinal microbiota. Other properties of LF, such as antistress, hypotensive and analgesic effects, are equally important. All of these effects of LF positively affect many aspects of our health, playing a particularly important role in the development of newborns and infants, as well as in older patients treated for a variety of infections (including bacteremia and sepsis) and during convalescence after serious diseases, surgery or chemotherapy [60,67,68,70,73,74,75,76,77,78]. LF may be regarded as a “biological drug” which is active by systemic (oral, intravenous, intramuscular and subcutaneous) and topical (on mucous membranes and skin) administration [60,61,62,64,65,66,67,68,70,73,74,75,76,77,78,79,80,81]. An overview of LF activity is shown in Figure 3.

LF inhibits the development of infections in both a direct and indirect manner. Direct effects of LF on microbes include: • binding iron ions and removing them from the microbe growth environment; • damage to cell structures (mainly cell membranes and walls and mitochondria), which leads to osmotic disruption and limited nutrition, resulting in microbe cells being weakened or killed; • induction of apoptotic death of infected cells, which restricts the spread of infection; • inhibiting pathogen adhesion to epithelial cells, which prevents the development of infection; • enzymatic destruction of virulence factors (e.g., enzymes and receptors) produced by pathogens; • inhibiting proton pumps (both H^+^/K^+^ ATPases and V-ATPases); • inhibiting the formation of biofilms on the surfaces of affected epithelia, implants, medical prostheses, etc.; • forming reactive oxygen species, which signal the inflammatory process and are toxic to microbes; • promoting growth of commensal physiological microflora on the skin and mucosa surfaces (these microbes compete with pathogenic or potentially pathogenic microbiota, thus restricting their growth) [58,60,67,68,70,76,79].

Indirect effects occur through regulation of immune system activity by strengthening it at early stages of infection (to accelerate the elimination of infectious agents and heal damaged areas) and weakening it at later stages (to facilitate the restoration of homeostasis).

LF regulates the maturation of T and B lymphocytes, the activity of NK cells, granulocytes, macrophages, dendritic cells and other immune cells, and the production of cytokines, metalloproteinases, oxygen free radicals and other inflammatory agents. Moreover, it accelerates the repair of damaged tissues and healing wounds [75].

LF has an extremely important role in the gastrointestinal tract, which is particularly important in newborns as well as in the elderly and active athletes. It stimulates growth, accelerates maturation and has a protective effect on intestinal tissues—epithelial cells and immune cells of lymph nodules—increases the secretion of digestive enzymes, strengthens the physiological intestinal microbiota, inhibits toxin production and the proliferation of pathogenic microorganisms, and seals tight junctions between epithelial cells, resulting in reducing intestinal permeability. In addition, LF inhibits chronic inflammation and the development of polyps, which represent an early stage of intestinal cancer [80,81,82].

Evidence from preclinical and clinical studies indicates that bovine-milk-derived LF has good bioavailability and a good safety profile with no serious side effects, both after systemic and topical use in humans and animals [73,77,78,83,84]. The protein is widely available and relatively cheap because it is isolated from milk in a well-established, standardized, large-scale manufacturing process [73,85]. The application of bovine LF has been approved by various agencies, such as the European Food Safety Authority (EFSA) in Europe and the Food and Drug Administration (FDA) in the US [86,87]. Of importance, despite concerns regarding possible enzymatic degradation of LF in the gastrointestinal tract, oral administration of LF in rats elicits similar intracellular signaling to LF given intravenously [88]. More importantly, buccal administration of LF to healthy volunteers resulted in regulation of their immune status [89,90].

## 4. Lactoferrin as Supportive Therapy in Antibiotic Treatment

Adverse side effects may occur during antibiotic therapy or combinatorial therapy with antibiotics with other types of drugs. The most common side effects of antibiotics are diarrhea, nausea, headache, intestinal inflammation, thrush, aphthae, rashes and vaginal mycosis. The side effects of excessive or non-selective antibiotic use are caused by elimination of both physiological and pathogenic bacteria. As a consequence, disruption to the composition of the natural intestinal and urogenital microbiota occurs. A therapy with single classical antibiotics may not be sufficient to cure some infections, so protocols involving several antibiotics with a different killing spectrum must be used. Such an approach increases the undesirable effects of the therapy. BC, LF and other BC-derived biocomponents can alleviate the side effects of antibiotic therapy but also may enhance its efficacy by direct and indirect (via immune system stimulation) antimicrobial effects. These natural products have a multi-directional effect on pathogenic microorganisms, so antibiotic resistance does not develop.

### 4.1. Antibiotic Treatment in In Vitro and Animal Models

In a mouse model, inflammatory foci in lungs of *Mycobacterium tuberculosis*-infected animals hamper the penetration of therapeutics to infected sites and bacteria destruction. Oral recombinant human LF (rhLF) was used in C57BL mice as an adjuvant therapy with ofloxacin fluoroquinolone [91]. Histological analysis revealed that treatment with LF enabled penetration of the antibiotic to the pathologic sites populated with activated macrophages. The increased antibiotic penetration also preserved endothelial cell integrity. In addition, the phenotypes of macrophages showed an increased M-2-like pattern.

*Entamoeba histolytica* is a dangerous parasite that attacks the liver and intestine, often causing dysentery by perforating the large intestine. Metronidazole is applied for the treatment of amoebiosis but produces toxic effects (such as nausea and a bad taste in the mouth) in patients. Hamsters infected with amoeba that were given bLF intragastrically (2.5 mg/100 g b.w.) for 8 days showed no symptoms of the disease, and amoebic liver abscess was present in less than 1% of the animals, compared with 63% in controls. In addition, the liver function and blood cell parameters were restored to almost normal levels [92].

Cystic fibrosis (CF) is often accompanied with infections by the opportunistic pathogen *Pseudomonas aeruginosa*. Inhibition of its elastase enzymes and iron uptake by its siderophores—pyoverdins—could be valuable therapeutic targets [93]. The elastase produced by *P. aeruginosa* causes a rapid release of iron from transferrin and its uptake by pyoverdins, but LF is not a source of iron for these bacteria [94]. This suggests the possibility of using LF to treat CF patients. These facts prompted clinicians to use exogenous LF for treatment of CF patients, since endogenous LF in the airways of CF patients is proteolytically cleaved by cathepsin activity in *P. aeruginosa* [95]. In vitro experiments were performed to evaluate a potential, concerted effect of LF and antibiotics on reducing bacterial colony forming units (CFUs) numbers in an in vitro cell line model [96] and sputum from CF patients [97]. For these studies, ALX-109—a combination of apo-bLF with hypothiocyanite (OSCN^-^—a bactericidal agent)—an experimental drug developed by Alaxia (Lyon, France), was used. In a model applying airway epithelial cells from CF patients infected with *P. aeruginosa*, ALX-109 was used in combination with tobramycin or aztreonam [96]. It appeared that ALX-109 alone reduced bacterial biofilms and showed an additive effect with tobramycin in reducing bacterial CFUs and enhanced the ability of aztreonam to reduce *P. aeruginosa* biofilm. The authors concluded that a combination of ALX-109 with antibiotics, in the form of an inhalatory aerosol, could be beneficial in decreasing bacterial infection in the airways of CF patients. Such an assumption was supported by another study in which sputum from CF patients was treated with ALX-009 (another combination of apo-bLF with hypothiocyanite) alone or combined with tobramycin [97]. ALX-009 alone demonstrated a bactericidal action on the *P. aeruginosa* in the sputum samples more potent than that of tobramycin, but this effect was even stronger when combined with tobramycin.

Bovine LF saturated in 85% with Fe^3+^ (holo-bLF) prevented the vegetative cell growth and toxin production of a clinical strain of *Clostridium difficile* in triple-stage chemostat gut models [98]. The glass vessels were inoculated with human feces and spiked with *C. difficile* spores, holo-bLF or apo-bLF (saturated in 1% with Fe^3+^), and clindamycin for simulation of *C. difficile* infection (CDI). Apo-bLF was ineffective. The authors concluded that LF may be a proper addition to the standard prevention and treatment of CDI in patients in long-term care. CDI develops after treatment with antibiotics and is a major cause of chronic, debilitating diarrhea in hospitals and care facilities. Hospital strains of this bacterium are often antibiotic-resistant, making treatment much more difficult. The results are consistent with the observation in a clinical trial that bLF reduced the incidence of post-antibiotic diarrhea in a long-term-care patient [99].

In in vitro tests, bLF and other iron chelators were tested against various strains of *Cryptococcus*, an opportunistic yeast that causes dangerous infections in immunocompromised patients. LF showed a synergistic effect with amphotericin B against all yeast strains tested, but this effect was not mainly due to iron chelation, but to other LF activities that were enhanced in the presence of the antibiotic [100].

### 4.2. Antibiotic Treatment in Clinics

In a case report with 26-year-old women suffering from post-influenza otitis media infection, no therapeutic benefit was obtained after treatment with antibiotics and steroids (Atecortin and Dicortineff) [101]. The pathogens were *Staphylococcus homis* and *Staphylococcus epidermidis*. A specific bacteriophage therapy (3 weeks) was also not completely effective. However, oral application of bLF (50 mg daily for 7 days, with two-week intervals), led to a full recovery, associated with increased myelopoiesis and elevated serum LF concentration.

Tooth extraction is often associated with post-extraction complications, even with application of antibiotics. A total of 111 patients with local post-extraction complications treated with antibiotics were enrolled in a clinical study [102]. The patients were divided into three groups taking (A) amoxicillin and clavulanic acid (2 g/day for 6 days), (B) the antibiotics plus *Bifidobacterium longum* (a probiotic) plus bLF and (C) a placebo group. The degree of complications was recorded on days 7, 14 and 21 after the extractions. The results showed that pain was present for 48%, 30% and 71.4% of the respective groups, with similar differences in the mean numeric rating scores. Two patients from the placebo group experienced dry socket. Nine patients from the antibiotic only group and one patient from the antibiotic and probiotic/LF group had intestinal distention. Diarrhea was registered in three patients from the antibiotic alone group but was absent in the other groups.

Antibiotic-associated diarrhea (AAD) can result from hospital-acquired infections. In a clinical, randomized, double-blind study involving tube-fed, long-term-care adult patients with antibiotic-associated diarrhea, rhLF was orally applied for 8 weeks [99]. It appeared that fewer patients in the LF-treated group suffered from diarrhea. However, in another prospective, randomized, double-blind, placebo-controlled, single-center study, no therapeutic benefit of bLF application in children (n = 156) with AAD was found [103].

In several clinical trials, LF was included in therapeutic protocols to prevent side effects of antibiotic therapy in *Helicobacter pylori* infection. In a clinical trial involving children, all patients were treated for 4 weeks with a combination of omeprazole, amoxicillin and clarithromycin (the therapeutic regimen also for group A) [104]. Some of the patients received, in addition, a probiotic Probinul-Cadigroup containing LF (group B). Health status was evaluated at the end of the treatment. The results of the treatments were as follows. Epigastric pain occurred in 17.6% of group A versus 5.8% of B group patients, nausea in 8.8% of group A versus 2.9% of group B, diarrhea and vomiting in 5.8% and 23.5%, respectively, of group A versus none in group B. A total of 2.3% of patients tested negative for *H. pylori.* In conclusion, the addition of LF to the therapeutic protocol decreased the occurrence of the described side effects.

In a similar study on adult patients (n = 206), bLF and probiotics were used to improve therapeutic efficacy and lower side effects of standard antibiotic therapy (esomeprazole, clarithromycin and amoxicillin) during an 8-week treatment [105]. At the end of the treatment, 84.95% of patients showed negative results for *H. pylori* in the group treated with antibiotics only and 92.0% in the group given, in addition, LF + probiotics. Moreover, more patients in the antibiotic group reported side effects. In conclusion, better eradication of infection and limitation of side effects were registered thanks to the inclusion of LF and probiotics in the triple antibiotic therapy for *H. pylori.*

A prospective, randomized study was also performed on 76 patients after failure of a first, conventional quadruple therapy [106]. All patients were treated with a combination of ranitidine bismuth citrate, esomeprazole, amoxicillin and tinidazole for 7 days. The therapeutic regimen was supplemented in one group with bLF (400 mg daily). One month after the therapy, an endoscopy and the ^13^C-urea breath test (UBT) were performed to evaluate the efficacy of the treatment. Eradication *H. pylori* in the bLF group was 94.3% (33/35) and in the control group was 88.6% (31/35), as assessed by UBTs and histological analysis. Side effects were recorded in 29.4% of patients in the antibiotic alone group versus 17.6% in the patients taking LF supplementation.

A recent parallel RCT included 400 adult patients with *H. pylori* infection [107]. They were randomized into four equal groups: (A) proton-pump-based triple therapy (PpTT) involving proton pump inhibitors: esomeprazole plus antibiotics: amoxicillin and clarithromycin for 2 weeks, (B) esomeprazole plus amoxicillin for 5 days and then esomeprazole plus metronidazole and clarithromycin 10 days, (C) PpTT as in group A plus bLF in 200 mg sachets (overall daily dose 400 mg) for 2 weeks and (D) sequential therapy as in group B plus bLF for 2 weeks, as above. Bacterial eradication was significantly better in both groups treated with LF (percentages of eradication for groups 1, 2, 3 and 4 were 70.3%, 82.8%, 85.6% and 94.5%, respectively). The authors made no mention of differences in the frequencies of adverse side effects of these therapies (dizziness and headache, fatigue, nausea, taste disturbance and colonic distension were observed).

LF, in addition to its antimicrobial effect, exhibits IPP-like activity, inhibiting both H+/K+-ATPase and V-ATPase. The protein was shown to be more active at acidic pH and had a good safety profile [79,108]. This LF activity may further enhance the efficacy of classical therapy used in *H. pylori* eradication.

## 5. Bovine Colostrum and Lactoferrin as a Supportive Therapy in NSAID Therapy

NSAIDs are effective in the management of pain, fever and inflammation occurring in a variety of clinical conditions. Long-term use of these drugs also protects against heart attack, so they are commonly prescribed by physicians. NSAIDs are among the most widely used drugs, particularly to treat common colds. The most frequently used NSAIDs include acetylsalicylic acid, phenylbutazone, indomethacin, diclofenac, ibuprofen, naproxen, nabumetone, meloxicam and piroxicam. However, these drugs cause numerous side effects in tissues and organs, mostly involving gastrointestinal, renal, cardiovascular, hematological, pulmonary, liver and neurological damage. NSAIDs may also cause allergic reactions [1]. The most common adverse effects following application of NSAIDs, are micro- and macro-damage of gastric and intestinal mucosa. This kind of damage leads to a weakening of the mucosal–blood barrier and increased permeability for food debris, microorganisms and their toxins, development of chronic inflammation, blood loss due to vascular damage, consequent scar formation, and stenosis or perforation of the gastrointestinal wall. Serious complications occur in about 2% of individuals taking NSAIDs.

Colostrum can prevent and treat the described changes, as it contains numerous components, including the growth factors IGF-1, TGF, VEGF and EGF, as well as heat shock protein 70 (Hsp70) with a protective function for cells (HSP protects the integrity of cell membranes under stress conditions). These factors stimulate the growth and regeneration processes of the gastrointestinal epithelium. In addition, regulation of epithelial cell apoptosis and expression of the adhesion molecule ICAM-1 and the cell junction proteins zonula occludens 1 (ZO-1) and claudin may also be important [109,110]. An effective supportive therapy during treatment with NSAIDs could help to maintain the doses of therapeutics and duration of therapy, with the least possible health burden to a patient. Below, a series of investigations are presented, proving the protective effects of BC and its components in experimental animals treated with NSAIDs and experiencing drug injury.

### 5.1. NSAID Therapy in Animal Models

Bovine LF in oral spray was used as a therapeutic with oral piroxicam in caudal stomatitis in cats [111]. Scoring for clinical symptoms was monitored and oral mucosal biopsies were taken for histological examinations. The effects of a combined administration of oral piroxicam and spray with LF, compared with piroxicam treatment alone, showed a better effect on reduction in oral lesions, correlated with a decrease in the number of macrophages and improvement of clinical symptoms, quality of life and weight gain.

In turn, rhLF was used to prevent NSAID-induced bleeding from the gastrointestinal tract in rats and mice [112]. Acute and chronic models involved application of indomethacin, naproxen and diclophenac. Orally administered hLF prevented drug-induced bleeding and inflammation, as well as myeloperoxidase activity, correlated with inhibition of neutrophil migration to the intestine. The action of hLF was not dependent on prostaglandins and nitric oxide metabolism, and LF did not interfere with drug activity.

In a similar study, NSAID-induced gastrointestinal tract damage was treated with C lobes of bovine LF [113]. The application of LF C-lobes after NSAID application partially reduced the drug-induced injuries to 47–70%, but the combined administration with the drugs ameliorated the injuries more significantly. Crystallographic methods showed NSAIDs binding to C lobes with a high affinity.

The protective effects of LF on NSAID-induced enteropathy were also investigated in rats given diclophenac by the intragastric route daily for 14 days [114]. LF, *Bifidobacterium* or a combination of LF and *Bifidobacterium* were administered every time, 1h before each diclophenac dose. The following parameters were evaluated after completion of the study: histological analysis of ileum, myeloperoxidase and malondialdehyde levels, expression of Toll-like receptor 2 (TLR2) and TLR4, activation of signaling molecules MyD88 and NF-κB p65, and blood hemoglobin and fecal calprotectin levels. As expected, diclophenac caused intestinal damage and changes in several parameters, such as overexpression of TLRs, MyD88 and NF-κB p65, increased fecal calprotectin and lowered blood hemoglobin. Although beneficial effects of single treatments were observed, the best results were obtained regarding the prevention of intestinal pathology and changes in other parameters using the combinatory treatment of LF and *Bifidobacterium*.

Another rodent investigation also demonstrated the efficacy of BC in protecting the stomach and intestine from damage induced by NSAIDs [115]. Application of 0.5 or 1 mL BC in rats, prior to indomethacin administration, reduced gastric damage by 30% and 60% respectively. A control milk preparation was significantly less effective. On the other hand, an addition of BC to the drinking water (10 % *v/v*) protected the mice from damage to the small intestine (shortening of intestinal villi). It could be possible that TGF-β, one BC component, which showed similar efficacy to BC, contributed to BC activity. In addition, in vitro tests showed that BC increased the proliferation and migration of intestinal epithelial cells of RIE-1 and HT-29 lines. According to the authors, BC may be a valuable agent to protect and treat damage after the application of NSAIDs in ulcers in the stomach and intestine of other etiologies.

In similar trials, the same research team confirmed the protective effect of BC in enteropathy after NSAID treatment [116]. In in vitro tests, using human and rat intestinal and gastric epithelial cells, the addition of BC or egg yolk powder increased the cells’ ability to proliferate and migrate three-fold. When the preparations were administered to mice with intestinal damage caused by indomethacin and rats with chemically induced colitis, they reduced gastrointestinal epithelial damage by 30–60%, and the effect was better when both preparations were used together.

In a recent study by Playford et al., in a rat model of gastric mucosa damaged by indomethacin and the concomitant stress of short immobilization, BC given orally (14 mg) attenuated the toxicity of the procedure, and its activity depended on the quality of the commercially available formulation used (reduction in macroscopic epithelial damage by 86% vs. 48% for the most and least active products, respectively) [117].

### 5.2. NSAID Therapy in Clinics

In a human, placebo-controlled trial involving 15 volunteers [118], orally given hLF was used for protection against NSAID-induced gastroenteropathy. The rise in epithelial permeability in the stomach and small intestine was measured with a non-invasive lactulose/rhamnose test as a parameter of drug-induced mucosal damage. The patients were treated with indomethacin in three doses, preceded by an LF drink. A placebo group was also included in the study. Small intestine permeability in the placebo group was significantly higher compared to the group taking LF. However, there was no difference in the gastroduodenal permeability between the groups.

Similar protective activity was confirmed for BC in another placebo-controlled clinical trial [48]. The study included healthy volunteers (n = 7) who took indomethacin for a short period of time (5 days) and patients (n = 15) who took NSAIDs regularly for a long period of time to treat various conditions. Both volunteers and patients ingested BC (125 mL three times daily) for 7 days. At the end of the testing period, intestinal permeability was determined with the lactulose/rhamnose test. Among the volunteers, there was a three-fold increase in intestinal permeability in the control group taking whey protein but not in the BC group. In the patients, intestinal permeability was low at the baseline and was not altered after BC application. According to the authors, this may have been the result of adaptation of the gut to the long-term use of NSAIDs.

## 6. Bovine Colostrum and Lactoferrin as Supportive Therapy in Steroid Treatment and Psychophysical Stress

Natural steroids (e.g., cortisol, cortisone and corticosterone) are secreted in the body by the adrenal cortex, depending on the diurnal rhythm. They affect the metabolism, neural, circulatory and immune systems. Endogenous steroids are essential during adaptation to stressful situations, so their concentrations increase during stress, leading to adverse consequences of chronic stress, such as cardiovascular diseases and immune deficiencies (among other things, stress modulates intestinal secretory IgA/sIgA/ levels and Th1-mediated immune response) [7,8,9,10]. Widely used in medicine, as analgesics and anti-inflammatory agents, synthetic steroids are easily absorbed in the gastrointestinal tract, so are usually administered orally, but they can also be inhaled or sometimes applied intravenously. The use of steroids may be associated with the development of life-threatening side effects, some of which include immunosuppression, hypertension, impaired glucose tolerance or diabetes, decreased blood potassium levels, adrenal insufficiency, weight gain, stretch marks and acne, muscle weakness, gastritis or stomach ulcers, emotional disturbances (depression, emotional vacillation, cognitive impairment and sleep disturbances), osteoporosis, bone fractures and cataracts [4,119,120].

### 6.1. Steroid Therapy and Endogenous Stress in Animal Models

Thus, immunosuppression, associated with undesirable consequences, may result from steroid treatment [119] or elevation of endogenous steroids under stressful conditions [120]. Interestingly, these effects can be counteracted by administration of LF, as demonstrated in animal experimental models. One also has to keep in mind that exogenously administered LF affects steroid metabolism. An intravenous injection of LF induces a strong rise in serum corticosterone, which does not occur in adrenalectomized mice, and results in a rapid elicitation of myelopoiesis [121]. A high content of neutrophils in the circulation, following LF injection may explain the protective effects of LF against bacterial infection. The effects of LF could be blocked by Mifepristone, a blocker of steroid receptors.

Long-term (5 h daily for 5 days) immobilization (psychic stress) resulted in a suppressed humoral and, to a higher degree, a cellular immune response in mice [122]. The suppression of the humoral immune response, measured by the number of antibody-forming cells to SRBCs, and the cellular immune response, determined by a delayed-type hypersensitivity response to ovalbumin, were reversed by applying bLF to drinking water. On the other hand, LF lowered an elevated delayed-type hypersensitivity in a short-time (5 h) immobilization test. Of importance, the lymphocytes from mesenteric lymph nodes of these mice spontaneously produced higher levels of TGF-β, an immunosuppressive cytokine. So, the effects of LF on chronic and short-term stress are regulatory.

The effects of oral bLF administration on parameters of innate immunity in intestinal tissue differed in mice subjected to short or chronic immobilization stress. In one study, BALB/c mice were treated orally with three increasing doses of bLF, followed by a short, acute (1 h) immobilization stress [123]. The IgA and IgA-associated proteins were measured in the proximal versus distal small intestine. LF in stressed mice induced higher levels of IgA and sIgA and higher expression of polymeric Ig receptor (pIgR), IL-4 and IL-6 in proximal intestine tissue. Likewise, in the distal intestine, higher levels of total IgA, α-/J-chain and pIgR proteins, as well as expression of the α-/J-chain and IL-4, were found. In addition, compared to the control (unstressed, LF-untreated mice), plasma corticosterone levels increased in stressed/LF-treated mice and non-stressed mice treated only with LF.

On the other hand, the same authors demonstrated downregulatory effects of LF in mice subjected to a chronic immobilization model [124]. In this investigation, the mice were subjected to 7-day immobilization stress, followed by bLF treatment. Plasma corticosterone and presence or expression of IgA-associated molecules and interleukins in the distal intestine were measured. LF suppressed the stress-elevated concentrations of the total antibody levels, α-chain, pIgR and IL-6 expression. Additionally, the corticosterone levels in mice treated with the highest dose of LF (5 mg daily) increased in all groups, in accordance with other data [121]. In summary, the results on LF effects in mice subjected to psychic stress revealed the immunoregulatory effects of the protein, depending on duration, intensity of stress and time of LF treatment, and indicated a regulatory effect of LF in maintaining homeostasis under stress.

Transport of captive animals also generates stress [125]. Six dolphins received bLF in their feed before 6 h transportation. Five dolphins served as a control group. The transport elicited increased concentration of serum cortisol, lymphopenia, eosinophilia and mild neutrophilia, reflecting a stress response. Among the studied parameters, the administration of LF lowered only the level of eosinophils. The application of bLF (100 mg/kg b.w.) in the immobilized rats also affected plasma glucose levels in the oral glucose tolerance test and lowered plasma corticosterone levels [126].

Importantly, LF can also abolish a perception of stress by acting on the central nervous system (CNS), as confirmed in animal studies [127,128]. LF counteracted the consequences of separation of 5–8-day-old rats from their mothers [128]. The rat pups were injected i.p. with bLF (10 mg/kg b.w.) or bovine serum albumin as a control, 30 min before separation. Distress activity was determined by recording body movements or ultrasonic vocalizations. LF significantly suppressed distress activity after only 5 min of separation. The effects of LF were reversed by opioid antagonists and nitric oxide synthase (NOS) inhibitors. In an adult rat model, i.p.-injected bLF (100 mg/kg) reduced stressful behaviors in freezing and maze tests. LF effects in the CNS were opioid- and NO-mediated [127].

One animal study showed an immunoprotective effect of LF during synthetic steroid therapy. In an oral candidasis mouse model, the animals were immunosuppressed with prednisolone 1 day before and 3 days after infection with *Candida* [129]. Such treatment resulted in lowering peripheral blood lymphocytes on day 1 and cervical lymph node cells on days 1, 5 and 6. Feeding of the animals with bLF counteracted the decrease in blood lymphocytes on day 1, and a rise in lymph node cells numbers was accompanied with a decrease in *Candida* colony-forming units in the oral cavity. In ex vivo tests, increased production of IFN-γ and TNF-α by lymph node cell cultures, stimulated by heat-killed *Candida* on day 6, was also found. In this study, an increased production of IL-12, IFN-γ and TNF-α by ConA-stimulated regional lymph node lymphocytes from LF-treated mice inversely correlated with *Candida* load in the oral cavity. As the authors concluded, the protective effect of LF was due to an increase in the number and activity of leukocytes.

Another study in a rat model showed a beneficial effect of bovine whey extract enriched with six growth factors on the healing of experimental incisional wounds [130]. The rats were systemically administered methylprednisolone, resulting in a 15% loss of body weight and 42–44% deterioration in wound healing rates. Wound histology of steroid-treated rats was characterized by an absence of cellular infiltrate and granulation tissue. Whey extract application to the wounds increased the cellular infiltrate and fibroblast activity, but not the inflammatory scores for the wounds in both normal and steroid-treated rats.

### 6.2. Endogenous Stress in Clinics

LF may also reduce stressful conditions in humans, as shown in studies on students [131,132]. In a single-dose, double-blind, placebo-controlled cross-over RCT, oral bLF (0.8 g) regulated the activity of the parasympathetic and sympathetic neural systems in female college students (n = 16) who solved a calculation test [131]. In another placebo-controlled cross-over study, orally administered bLF reduced the mental stress reaction in the CNS and autonomic nervous system and restored immune response in adult subjects, who showed a stress reaction to the calculation work [132]. The normalization of brain waves, improvement of scores in psychological tests and an increase in salivary sIgA were observed.

Intense physical exertion during training or sporting competitions, especially in unfavorable environmental conditions (high humidity and high temperatures), causes severe psychophysical stress in the organism. It is one of the factors that worsens the function of the digestive tract. Other adverse factors include reduced blood flow in the gastrointestinal wall, poorer absorption of water, electrolytes and glucose, changes in intestinal motility, and hormonal and immune system imbalances. Intestinal disorders are found in more than half of marathon and ultra-endurance runners. At the cellular level, damage to the intestinal epithelial cells (enterocytes) is found, specifically with damage to the tight junctions—the protein structures that connect the individual cells. Damage to intestinal barrier integrity leads to increased permeability of the intestinal wall to substances contained in its lumen: undigested food components and microorganisms and their toxins (e.g., LPS) [53,54,133].

BC supplementation may protect intestinal integrity in people who exercise intensively, as confirmed in several RCTs [134,135,136,137]. Eight actively training healthy volunteers participated in the double-blind cross-over RCT [134]. BC formulation with carnosine and zinc (20 g daily for 2 weeks), administered during intensive exercise, reduced increased intestinal permeability (as measured by the lactulose/rhamnose test). In an in vitro test, BC increased the tightness of the intestinal epithelium Caco-2 and HT-29 cell lines. Increases in Hsp7 protein and total occludin and decreases in phosphorylated ZO-1 and occludin, as well as decreases in proapoptotic proteins (Bax and caspase-3 and -9), were also observed. Occludin and ZO-1 are proteins that form tight junctions between cells.

In another, double-blind and open-label (cross-over) two-phase RCT involving 16 athletes, a commercial BC preparation obtained within 2 h of calf delivery (1 g daily for 3 weeks) also reduced intestinal permeability, as measured by urine lactulose/mannitol ratio and lower levels of zonulin in stools, reflecting the effective reversal of inappropriately increased intestinal permeability [135]. In a similar, later double-blind RCT, the same authors demonstrated the efficacy of 2 h and 24 h BC preparations (1g daily for 3 weeks) in 31 healthy athletes (as measured by the lactulose/rhamnose test). Only these early BC preparations (not 72 h BC preparations) were effective in reducing intestinal permeability during athletic training [136].

In a further double-blind cross-over RCT involving 18 athletes, a commercial BC preparation (20 g daily for 2 weeks) reduced intestinal permeability (as measured by the lactulose/rhamnose test) that had increased after an intensive run for 1 h. In addition, a smaller increase in serum protein I-FABP, a marker of intestinal epithelial damage, was reported [137].

The benefits of BC and LF, as supportive care in antibiotic, NSAID and corticosteroid use, as well as in stress (effects of endogenous steroids), in in vitro models, animals and humans are summarized in Table 1.

## 7. Conclusions

Results from various animal models and clinical trials have demonstrated that oral BC and LF and LF-derived peptides ameliorate undesirable side effects of therapeutic treatments but also enhance the efficacy of therapeutic procedures with antibiotics, NSAIDs and steroids.

BC and LF are effective when applied alone and together with the therapeutics contained in diet supplements and nutraceutics or applied with probiotics. The desirable effects of BC and LF administration stem from their multiple physiological properties, such as promotion of T and B cell maturation, elicitation of myelopoiesis, the effect on hypothalamus–pituitary–adrenal axis function, regulation of iron metabolism, inhibition of pathogenic microbes, stimulation of beneficial intestinal microbiota growth and stabilization of intestine mucosal membranes.

A wide assortment of commercial products containing BC and LF are available, ranging from food products to dietary supplements and cosmetics. Formulations with LF and colostrum are also used in animal nutrition as feed additives for farm animals, including fish. Based on the cited literature we do not hesitate to recommend the inclusion of BC and LF in various therapeutic protocols with accompanying adverse side effects. However, we are aware of some limitations of the presented results. They include, among others, the application of a variety of not well-defined and standardized LF and BC products. Their quality is influenced by many factors, from the source of the BC (season, breed, age of cows, feeding regime and time of acquisition after calving) to the production process of LF and BC powders (pasteurization and drying conditions: freeze-drying versus spray-drying) [24,55,85]. In addition, it is not possible to establish optimal average therapeutic doses for each preparation. The experimental protocols vary, depending on the category of patients, and may range from a few milligrams to several grams in the case of LF, being usually, on average, much higher for BC (grams) [11,14,15,16,17,18,19,20,22,23,44,45,46,47,48]. It must also be mentioned that both sorts of preparations are freely available on the market with manufacturer’s recommendations regarding their application.

In conclusion, the presented review of animal and clinical studies allows BC and LF to be considered natural, safe and valuable products that can be used for adjunctive therapy in the treatment of infections with antibiotics or inflammation with NSAIDs and corticosteroids. BC and LF can be also recommended for humans subjected to prolonged stress, especially in high ambient temperatures (soldiers and emergency services), patients after surgery and accidents, physically active people and training athletes.

## Figures and Tables

**Table 1:** Protective and therapeutic effects of whole BC and LF (bovine and human) as additive therapies in antibiotic, NSAID therapy, synthetic steroid therapy and psychophysical stress (generated by endogenous steroids). In vitro/ex vivo, animal and human studies are presented.

Model	Application of Colostrum Proteins	Therapeutic Laboratory or Clinical Effects	Reference
**Antibiotic therapy in in vitro and animal models**			
Mice infected with *Mycobacterium tuberculosis* and treated with ofloxacin fluoroquinolone	Orally applied rhLF	LF enabled penetration of the antibiotic to the sites of pathological disruption, increase in macrophage phenotype in the granulomas to M-2-like phenotype	Nguyen T.K.T. et al. (2022) [91]
Hamsters infected with amoebae	Intragastrically given bLF (2.5 mg/100 g mass) for 8 days	Prevention of disease symptoms, the liver functions and blood parameters return to normal levels	Ordaz-Pichardo C et al. (2012) [92]
Six clinical isolates of *P. aeruginosa* biofilm grown at the apical surface of human CF airway epithelial cells	bLF-hypothiocyanite combination (ALX-109) combined with tobramycin or aztreonam	ALX-109 alone reduced bacterial biofilm formation but did not disturb the biofilms already formed, enhancement of tobramycin and aztreonam ability to reduce biofilm formation and disrupt existing ones	Morceau-Marquis S. et al. (2015) [96]
Sputum from CF patients containing *P. aeruginosa* and *Burkholderia cepacia* complex (Bcc)	bLF-hypothiocyanite combination (ALX-009) alone or combined with tobramycin	ALX-009 alone had a bactericidal effect against *P. aeruginosa* but was more effective in combination with tobramycin	Tunney M.M. (2018) [97]
*C. difficile* infection in triple-stage chemostat gut model inoculated with human feces	Holo-bLF and apo-bLF instilled in the chemostat with simulated CDI, 7 days later clindamycin was instilled	Holo-bLF, but not apo-bLF, delayed growth of *C. difficile* and prevented toxin production	Chilton C.H. et al. (2016) [98]
*Cryptococcus* strains in in vitro cultures	bLF (and other iron chelators) with antifungal agents (among others, amphotericin B, fluconazole and itraconazole)	Significant synergy between LF and amphotericin B for all *Cryptococcus* strains, not primarily due to iron chelation but to other properties of LF that were raised in the presence of amphotericin B	Lai Y.-W. et al. (2016) [100]
**Antibiotic therapy in clinics**			
Case study; patient with ineffective treatment of post-influenza otitis with antibiotics and specific bacteriophages	Orally applied bLF (50 mg daily) for 7 days, with two-week intervals	Complete eradication of the inflammation, complete clearance of both bacterial strains (*Staphylococcus homis* and *Staphylococcus epidermidis*), increased myelopoiesis and elevated serum LF concentration, and full recovery of the patient	Weber-Dabrowska B. et al. (2006) [101]
Monocentric RCT; patients with tooth post-extraction complications; n = 111	Amoxicillin + clavulanic acid (#1) for 6 days after extraction versus the antibiotics + *Bifidobacterium longum* + LF (#2) and no antibiotic therapy (#3)	Pain at surgical site relief (pain was present in 48%, 30% and 71.4% of the respective groups #1, 2 and 3) Protection from diarrhea (was recorded in 5 patients in group #1 and in none of the patients in groups #2 and #3) and intestinal distention (in 9 patients in group #1 and 1 patient in group #2)	Barone A. et al. (2017) [102]
Double-blind RCT; tube-fed long-term-care adult patients with AAD; n = 30 (males and females)	Orally given (by gastric tube), rhLF (3 g daily) for 8 weeks versus antibiotics only	Fewer patients experienced AAD (including *Clostridium difficile* diarrhea) Reduced mean number of diarrhea days and the percentage of the study days with AAD Shorter time to first episode of ADD	Laffan A.M. et al. (2011) [99]
Randomized clinical trial; children (8.3 ± 3.4 years) treated for *Helicobacter pylori* infection with standard triple treatment; n = 68 (males and females)	Omeprazole + amoxicillin + clarithromycin (#1), additional probiotic + bLF (no dose specified) (#2)	Alleviation of antibiotic therapy side effects: epigastric pain in 17.6% versus 5.8% of patients, nausea in 8.8% versus 2.9%, vomiting and diarrhea in 5.8% versus 23.5% (group #1 versus group #2)	Tolone S. et al. (2012) [104]
Open, prospective, randomized study; adult patients treated for *H. pylori* infection with standard triple treatment; n = 206 (males and females)	Esomeprazole + clarithromycin + amoxicillin + bLF (400 mg daily) + probiotics for 7 days versus antibiotics only as control	Eradication of *H. pylori* in 88.6% of the LF + probiotics group and 72.3% of the control group, in ^13^C-urea breath test Fewer adverse side effects in the LF + probiotics group (in 9.5%) versus control group (in 49.6%)	de Bortoli N. et al. (2007) [105]
Prospective, randomized study; adult patients with persistent *H. pylori* infection, with peptic ulcer or gastritis with severe histological abnormalities failure of the first standard treatment schedule; n = 70 (males and females)	Ranitidine bismuth citrate + esomeprazole + amoxicillin + tinidazole + bLF (400 mg daily) for 7 days versus antibiotics only as control	Eradication of *H. pylori* in 94.3% of the LF group and 88.6% of the control group, in ^13^C-urea breath test and histological evaluation Adverse side effects in 17.6% of patients in the LF group and 29.4% of the control group	Tursi A. et al. (2007) [106]
Parallel randomized study; adult patients with *H. pylori* infection with standard triple treatment or standard sequential treatment; n = 400 (males and females)	Esomeprazole + clarithromycin + amoxicillin + bLF (400 mg daily) or esomeprazole + amoxicillin and then esomeprazole + metronidazole + clarithromycin + bLF (400 mg daily) for 15 days	*H. pylori* eradication in groups without LF: 70.3% and 82.8%; in groups with LF: 85.6% and 94.5% No differences in the frequency of side effects of therapy	Hablass F.H. et al. (2021) [107]
**NSAID therapy in animal models**			
Double-blind RCT, cats with caudal stomatitis treated with piroxicam; n = 13	bLF in oral spray (2 sprays, 3 mg/spray, 6 mg/cat) with oral piroxicam versus piroxicam alone	Better reduction in oral lesions closely correlated with decreased number of macrophages and improvement of clinical symptoms, quality of life and weight gain	Hung Y.-P. et al. (2014) [111]
Rats and mice with NSAID-induced increases in gut bleeding after treating with indomethacin, naproxen and diclophenac	Orally or intraperitoneally applied rhLF (30, 100, 200 or 400 mg/kg b.w.)	Prevention of drug-induced bleeding, inflammation, inhibition of myeloperoxidase activity and neutrophil migration to the intestine; LF activity is independent of prostaglandins and nitric oxide; LF does not bind to the NSAIDs or interfere with the NSAID biological activity	Dial E.J. et al. (2005) [112]
Mice with gastrointestinal damage after treatment with indomethacin, diclofenac, aspirin or ibuprofen	Orally applied C lobes (C-terminal half) of bLF prepared proteolytically (200 mg.kg b.w.)	Partial reduction in NSAID-induced injuries (gastropathy) by C lobes of LF applicated after NSAIDs, but better results with co-administration with NSAIDs; NSAID binding to C lobes was demonstrated	Mir R. et al. (2009) [113]
Rats with enteropathy after treatment with diclophenac	Orally given LF (100, 200 or 400 mg/kg b.w.) with *Bifidobacterium longum* versus *B. longum* alone	LF or *Bifidobacterium* alone were effective, but the combinatory treatment was more effective in reducing altered parameters in enteropathy, such as histological picture, MPO and MDA levels, TLR2 and TLR4 expression, activation of MyD88 and NFκB p65, blood hemoglobin and fecal calprotectin levels	Fornai M. et al. (2020) [114]
Rats and mice with gastro- and enteropathy after treatment with indomethacin; in vitro test	Rat model: Oral pretreatment with BC (0.5 or 1 mg) Mouse model: Addition of BC to drinking water (10% *v/v*)	Rat model: Reduction in gastric injury by 30% and 60%, respectively, for the doses Mouse model: Reduction in villus shortening in small intestine In in vitro test: Increased proliferation and migration of intestinal epithelial cell lines: human HT-29 and rat RIE-1	Playford R.J. et al. (1999) [115]
Mice with enteropathy after treatment with indomethacin Rats with chemically induced colitis	Mouse model: Oral pretreatment with BC (20 mg/kg b.w.) for 7 days Rat model: Oral pretreatment with BC (20 mg/kg b.w.) for 9 days	Reduced shortening of intestinal villi in mice by 34% and colonic damage in rats by 44–61% In in vitro test, 3-fold increase in epithelial cell (Caco-2, RIE-1 and AGS) proliferation and migration	Playford R.J., Garbowsky M., Marchbank T. (2020) [116]
Rats with gastric damage after indomethacin and short-term (3 h) restraint stress; in vitro test	Oral pretreatment with BC (14 mg/rat, 20 commercial BC products) in single dose 30 min. before indomethacin application and placement in restraint cages	Reduced macroscopic gastric injures by 48% and 86%, for least active and most active BC samples Reduced microscopic injures (histological ulcer score) by 15% and 74%, for least active and most active BC samples In in vitro test, increase in epithelial cell (Caco-2, RIE-1 and AGS) proliferation and migration	Playford R.J., Cattell M., Marchbank T. (2020) [117]
**NSAID therapy in clinics**			
A randomized cross-over, double-blind trial: Healthy volunteers treated with indomethacin for 5 days; n = 7 (males) Adult patients taking a substantial, regular dose of an NSAID for clinical reasons; n = 15 (male and female)	Healthy volunteers: Orally applied BC (125 mL, three times daily) with indomethacin for 7 days; Patients: Orally applied BC (125 mL, three times daily) for 7 days In both studies, whey protein as control	Healthy volunteers: 3-fold increase in gut permeability (lactulose/rhamnose test) in the control arm and no significant increase in permeability when BC was co-administered Patients: Initial gut permeability was low despite continuing with the drug, and was not influenced by co-administration of BC (possibly as an adaptation to long-term NSAID therapy)	Playford R.J. et al. (2001) [48]
A randomized cross-over dietary intervention; human volunteers given indomethacin versus placebo; n = 15 (males)	Orally applied rhLF (5 g in single dose) before indomethacin application	Small intestine permeability in lactulose/rhamnose test lower in LF group versus the placebo group	Troost F.J. (2003) [118]
**Steroid therapy and psychic stress in animal models**			
Mice under long-term (5 h daily for 5 days) and short-term (5 h) immobilization stress	0.5% bLF in drinking water	Restoration of DTH to OVA and HIR to SRBCs in long-term stress, reduced production of TGF-β by mesenteric lymph node lymphocytes Reduction in DTH in short-term stress	Zimecki M. et al. (2005) [122]
Mice under acute immobilization stress	Orally applied bLF (0.05, 0.5 or 5 mg) for 7 days before stress	Induction of higher levels of IgA, sIgA, pIgR, IL-4 and IL-6 in the intestine, higher levels of plasma cortisone in LF- and LF/stressed mice	Pena-Juarez M.C. et al. (2016) [123]
Mice under chronic (7-day) immobilization stress	Orally applied bLF (0.05, 0.5 or 5 mg) for 7 days before stress	Suppression of total antibody levels, α-chain, expression of polymeric IgR and IL-6	Cruz-Hernandez T.R. et al. (2021) [124]
Captive dolphins under 6h-transport as a source of stress	bLF (40 mg/kg b.w.) in feed before transportation	No effect on serum cortisol or lymphopenia, but lower eosinophilia in circulating blood	Noda N. et al. (2006) [125]
Rats under short immobilization stress	bLF (100 mg/kg b.w.) given i.p. before the stress	Stress significantly increased plasma glucose levels Plasma glucose levels in the oral glucose tolerance test were significantly decreased in the LF group Lowering increased plasma corticosterone levels during stress; no effect on insulin, epinephrine or glucagon levels	Maekawa Y. et al. (2017) [126]
5-8-day-old rat pups under maternal separation stress	bLF (100 mg/kg b.w.) i.p. 30 min. before the behavioral separation test	Suppression of distress activity (body movements and ultrasonic vocalizations), LF effects in CNS are opioid- and NO-mediated	Takeuchi T. et al. (2003) [128]
Adult rats under stress in freezing test and maze test	bLF (30 or 100 mg/kg b.w.) i.p.	Suppression of distress activity, LF effects in CNS are opioid- and NO-mediated	Kamemori N. et al. (2004) [127]
Mice with oral candidasis after immunosuppression with prednisolone Ex vivo tests in lymph node lymphocytes	0.3% bLF (equivalent of 0.5 g/kg b.w.) in drinking water	Prevention of decline in peripheral blood leukocyte number Increased antigen-specific production of IL-12, IFN-γ and TNF-α by cervical lymph node lymphocytes from LF-treated mice inversely correlated with *Candida albicans* load in oral cavity	Takakura N. et al. (2004) [129]
Methylprednisolone-treated rats with experimentally induced wounds	Bovine-milk-derived growth-factor-enriched preparation applicated to wounds	Promotion of cutaneous repair in normal and steroid-treated rats Increase in the cellular infiltrate and fibroblast activity, but not the inflammatory scores in wounds in both normal and steroid-treated rats	Rayner T.E. et al. (2000) [130]
**Psychic and psychophysical stress in clinics**			
Placebo-controlled cross-over study; adult subjects under mental stress when solving a calculation task; n = 24	Orally applied bLF (0.8 g) in single dose	Improvements in CNS, AUS and immune response: normalization of brain waves, improvement of scores in calculation tests, reduction in POMS scores (anger–hostility, vigor, fatigue, confusion) Increase in salivary sIgA level	Yoshise R.E. et al. (2010) [132]
Single-dose, double-blind, placebo-controlled cross-over RCT; college students who solved a calculation task; n = 16 (female)	Orally applied bLF (0.8 g) in single dose versus placebo (soy milk) as control	Normalization of the activity of parasympathetic and sympathetic neural system	Shinjo T. et al. (2018) [131]
Double-blind cross-over RCT; actively training healthy adult volunteers; n = 8 (males) In vitro test	Orally applied commercial BC preparation (20 g) plus zinc carnosine daily for 2 weeks versus isocaloric milk protein preparation as control	Reduced increased intestinal permeability (as measured by the lactulose/rhamnose test) In in vitro tests, increased the tightness of the intestinal epithelium Caco-2 and HT-29 cell lines Increase in Hsp7 protein and total occludin, decrease in phosphorylated ZO-1 and occludin, decrease in proapoptotic proteins (Bax and caspase-3 and -9)	Davison G. et al. (2016) [134]
Double-blind and open-label cross-over RCT; healthy adult athletes; n = 16 (males)	Orally applied commercial 2 h BC preparation (1 g) daily for 3 weeks versus isocaloric whey protein preparation as control	Reduced intestinal permeability (as measured by the lactulose/mannitol differential sugar absorption test) Lower levels of zonulin in the circulation, reflecting less damage to the intestinal epithelium Fewer cases of URTIs	Hałasa (2017) [135]
Double-blind RCT; healthy adult athletes; n = 31 (males and females)	Orally applied commercial 2 h BC preparation (1 g) daily for 3 weeks versus 24 h BC, 72 h BC and isocaloric whey protein preparation as control	Reduced intestinal permeability (as measured by the lactulose/mannitol differential sugar absorption test) only in 2 h BC and 24 h BC groups	Hałasa (2020) [136]
Double-blind cross-over RCT; healthy adult athletes; n = 18 (males)	Orally applied commercial BC preparation (20 g) daily for 2 weeks versus isocaloric protein preparation as control	Reduced intestinal permeability (as measured by the lactulose/rhamnose test) increased after an intensive run for 1 h Smaller increase in serum protein I-FABP, a marker of intestinal epithelial damage	March D.S. et al. (2017) [137]

AAD—antibiotic-associated diarrhea; apo-LF—iron-free LF; holo-LF—iron-saturated LF; bLF—bovine lactoferrin; CDI—*Clostridium difficile* infection; AUS—autonomic nervous system; CNS—central nervous system; CRP—C-reactive protein; DTH—delayed-type hypersensitivity; HIR—humoral immune response; I-FABP—intestinal fatty acid-binding protein; Ig—immunoglobulin; LF—lactoferrin; MDA—malondialdehyde; MPO—myeloperoxidase; NO—nitrogen oxide; NSAIDs—non-steroidal anti-inflammatory drugs; OVA—ovalbumin; i.p.—intraperitoneally; s.c.—subcutaneously; i.v.—intravenously; POMS—Profile of Mood States; RBCs—red blood cells; rhLF—recombinant human lactoferrin; RCT—randomized controlled clinical trial; sIgA—secretory IgA; SRBCs—sheep red blood cells; TLR—toll like receptors; URTIs—upper respiratory tract infections; ZO-1—zonula occludens protein-1; 2 h BC—BC preparation obtained within 2 h of calf delivery.

## Data Availability

No new data were created or analyzed in this study. Data sharing is not applicable to this article.
